# Exploration on the Approaches of Diverse Sedimentations in Polyphenol Solutions: An Integrated Chain of Evidence Based on the Physical Phase, Chemical Profile, and Sediment Elements

**DOI:** 10.3389/fphar.2019.01060

**Published:** 2019-09-18

**Authors:** Hao-zhou Huang, Bi Feng, Jun-zhi Lin, Sheng-yu Zhao, Hong-yan Ma, Hai-yan Liu, San-hu Fan, Zhen-feng Wu, Run-chun Xu, Li Han, Ding-kun Zhang

**Affiliations:** ^1^Pharmacy College, Chengdu University of TCM, Chengdu, China; ^2^Key Laboratory Breeding Base of Systematic Research and Utilization on Chinese Material Medical Resources Co-founded by Sichuan Province and Ministry of Science and Technology, Chengdu, China; ^3^Central Laboratory, Teaching Hospital of Chengdu University of Traditional Chinese Medicine, Chengdu, China; ^4^Sanajon Pharmaceutical Group, Chengdu, China; ^5^Sichuan Key Laboratory of Dairy Nutrition and Function, Chengdu, China; ^6^Jiangxi University of TCM, Nanchang, China

**Keywords:** polyphenols, Triphala, integrated chain, sediment, stability, association colloid

## Abstract

*Triphala* is a famous herbal formula originated in Asia and is popular in America. Due to the high abundance of polyphenols, its oral liquid is unstable and easy to cause precipitate, which results in the loss of activities. However, complex composition and unclear precipitation mechanism hinders the improvement of stability. In this study, the accumulation of precipitation in the storage and its effect on activity were investigated. Then, an integrated chain of evidence was proposed based on the physical phase, chemical profile, and sediment elements. The results showed that antioxidant activity decreased from *IC_50_* 115 to 146 μl before and after 90 days of storage, and the anti-fatigue activity decreased from 30.54 to 28.47 min. Turbiscan Lab Expert observed that particle size increased from 106 to 122 nm, and the turbiscan stability index increased from 0 to 14, which indicated that its stability is continuously decreasing. High performance liquid chromatography (HPLC) fingerprint coupled with multivariate statistical analysis identified that these chemical markers changed significantly, such as gallic acid, catechins, and ellagic acid. Loss of catechins tends to be involved in the formation of phlobaphene precipitation. The fact that the new-born ellagic acid in precipitation (0.47 mg/ml) is significantly higher than that reduced in solution (0.25 mg/ml) indicates that it is not only derived from colloid aging. Microscopic observation combined with energy spectrum analysis further confirmed the existence of the multi-precipitates. The crystalline precipitate is ellagic acid, and the other is phlobaphene. In conclusion, based on the evidence chain analysis, this study revealed a systematic change of the whole polyphenol solution system. It provides a novel perspective to understand the sedimentation formation of polyphenol solution, which is an important theoretical contribution to the preparation of polyphenol solutions.

## Introduction

Polyphenols are very common compounds in nature. It is a kind of complex phenolic secondary metabolites, widely distributed in common fruits and herbs, such as pomegranates, grapes, blueberries, *Sanguisorba officinalis*, *Terminalia chebula Retz*, *Rheum palmatum L*, and so on. Therefore, a great quantity polyphenols can be found in such marketed foods and drugs containing these fruits and herbs (Liu and Henkel, 2002). Polyphenols have been confirmed as various biological activities, including antioxidation (Mertens-Talcott et al., 2017), anti-inflammation (Oliviero et al., 2018), antibacterial (Smullen et al., 2007), and treatments of burns, mutations (Sharma et al., 2016), and cardiovascular diseases (Carluccio et al., 2003). At present, the development of foods, health products, and pharmaceuticals containing polyphenols has aroused wide concerns.

Polyphenols are often prepared into oral liquids for the good water solubility. However, these oral liquids are easy to precipitate during the storage period. The generation of sediment not only seriously affects the appearance and the contents of active components but also impairs the therapeutic effect and patient psychology. To avoid this problem, some clarification methods have been adopted, such as alcohol sedimentation, gelatin adsorption, and chitosan adsorption. Although these measures have been widely applied to remove some unstable particles in preparation process, the formation of sediment is still inevitable. It is primarily due to the indistinct mechanism of sedimentation formation.

*Triphala*, a famous prescription made up of three species of Southeast Asian tropical fruits *Phyllantus emblica L.*, *Terminalia chebula* Retz. and *Terminalia bellirica* (Gaertn.) Roxb., originated from India’s ayurvedic medicine. Modern studies have confirmed a variety of pharmacological effects, such as antioxidant (Bhattacharya and Agarwal, 2008), immunoregulation (Srikumar et al., 2007), antiradiation (Mano et al., 2005), antibacterial (Sabina and Rasool, 2008), and liver protection (Bhatnagar et al., 2015), and also revealed its good therapeutic effect on hyperlipidemia (Singhal et al., 2015), tumor (Chandran et al., 2015), and gingivitis (Pradeep et al., 2016). Due to its remarkable effect, it has been developed into various dosage forms (Jia et al., 2016), and its extracts are used as dietary supplements. In China, it has been developed into oral liquid through modern pharmaceutical technology, and called Triphala oral liquid (TOL). It is a very popular anti-fatigue product, especially in the students. However, ascribe to a large number of polyphenols (Avula et al., 2013), it is easy to form sediment during storage when stored for 1–2 months. Traditionally, the precipitation is attributed to phlobaphene, the oxidation and polymerization product of tannins. But it is not clear how the phlobaphene is produced and whether the sediment is only phlobaphene. To some extent, the lack of mechanism research has become a huge obstacle to improve storage stability.

**Figure 1:**
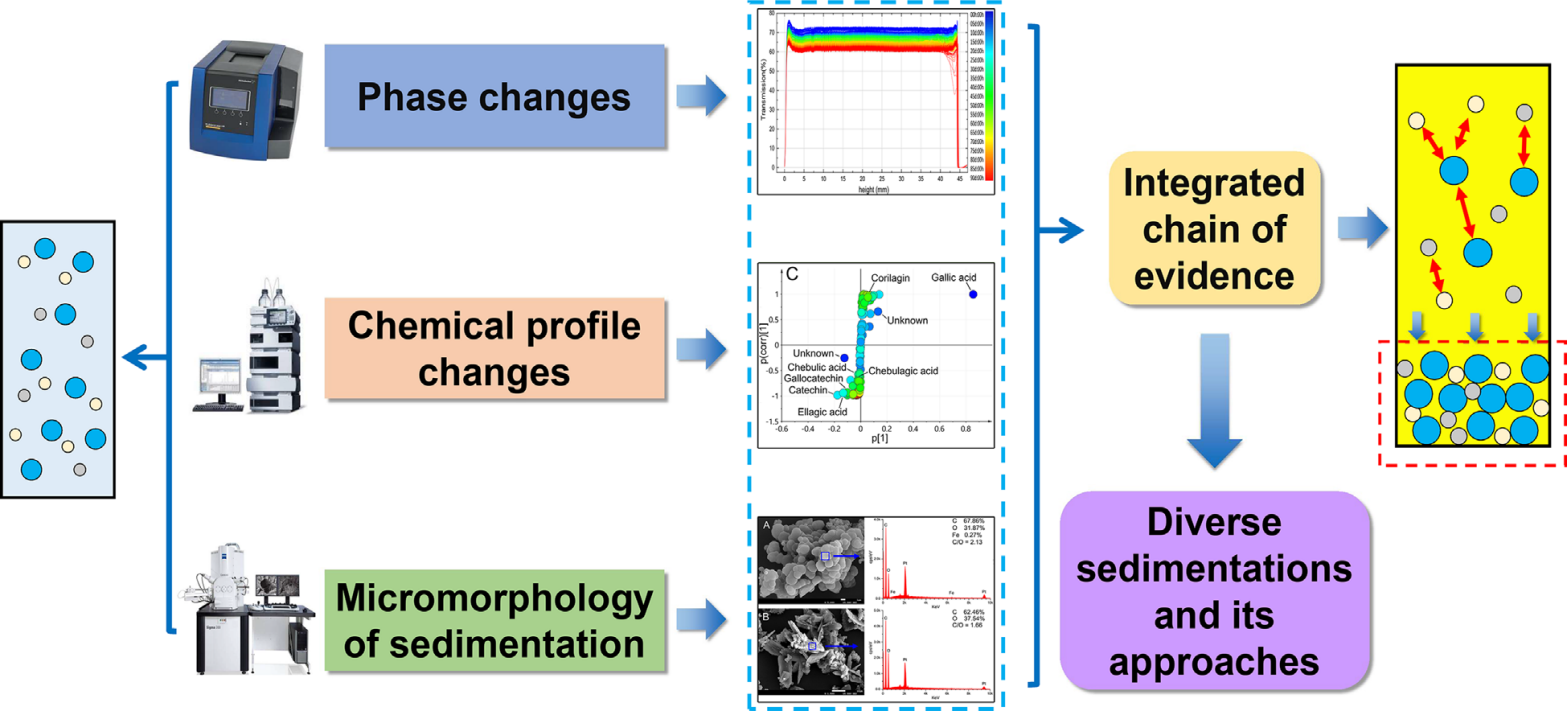
Flow chart of the manuscript.

The precipitation process is often accompanied by the phase changes, chemical profile transformation of the supernatant, and the aggregation and growth of sediment. It is a systematic change of the whole solution system. If we only focus on the chemical composition of the precipitate and neglect the synchronous changes in physicochemical properties of solution, it is difficult to reveal the microscopic mechanism of precipitation formation. In this manuscript, an integrated evidence chain research strategy was proposed based on the physical phase, chemical profile, and sediment analysis. The flow chart was shown in **Figure 1**. We hope this study can provide a novel perspective to understand the sedimentation formation of polyphenol solution and develop a corresponding stability strategy to ensure its efficacy. This is quite important for improving the stability of the decoction, oral liquids, and beverages. It will pique pharmaceutical and food development engineers’ interest and trigger research ideas controlling the quality of polyphenol solutions.

## Materials and Methods

### Ethics Statement

This study was conducted in strict accordance with the recommendations of the Guidelines for the Care and Use of Laboratory Animals of the Ministry of Science and Technology of China. The animal protocol was approved by the Committee on the Ethics of Animal Experiments of Affiliated Hospital of Chengdu University of Traditional Chinese Medicine (Approval ID: 2018BL-002).

### Materials and Chemicals

*Triphala* oral liquids were manufactured by Sichuan Huamei Pharmaceutical Co., Ltd., and the batch number was 1601001. Water was purified using a Milli Q water purification system (Millipore, Bedford, MA, USA). HPLC-grade methanol was purchased from Fisher Chemical (Pittsburg, PA, USA). HPLC-grade formic acid was obtained from Chengdu Kelong Chemical Factory (Chengdu, China). Anhydrous ethanol (analytical purity) was also bought from Chengdu KeLong Chemical Factory (Chengdu, China). Standard of 1,1-diphenyl-2-picrylhydrazyl (DPPH BR 97%R1701) was purchased from Duly Biotech Co., Ltd. (Nanjing, China). Standards of gallic acid (GA, no.4051109), epigallocatechin (EGC, no.14051611), epicatechin gallate (ECG, no.14050602), corilagin (CR, no.PRF7102406), gallocatechin (GC, no.4051109), catechin (C, no.14051508), epigallocatechin gallate (EGCG, no.14121608), gallocatechin gallate (GCG, no.14102009), ellagic acid (EA, no.PRF7101305), chebulagic acid (CLA, no.CHB171222), and chebulic acid (CA, no.AP8070902) were purchased from Chengdu Biopurify Phytochemicals Ltd. (Chengdu, China). The purity of the 12 standards was each above 98.0%. Magnesium standard solution (no. GSB04-1735-2004), calcium standard solution (no. GSB-SCD-002-2013), and iron standard solution (no. GSB04-1742-2004) were all received from National Non-ferrous Metals and Electronic Materials Analysis and Testing Center.

### Animals and Anti-Fatigue Experiment

Kunming mice, half male and female, weighing 18–22 g, were provided by the Laboratory Animal Research Institute of Sichuan Academy of Medical Sciences, (Permit No. SCXK (chuan) 2015-01). The animals were maintained under controlled conditions of temperature 20 ± 0.5°C, humidity 55 ± 5%, and with 12 h light and 12 h dark cycles. Thirty mice were randomly divided into three groups consisting of 10 animals in each. They were blank group, before storage group, and after storage (3 months) group, respectively. Time from swimming to sinking into water was recorded to evaluate the effect of precipitation formation on anti-fatigue function. The dose is 10 ml kg^−1^, and the administration time is 7 days.

### Preparation of Supernatant, Sediment Samples, and Standard Solutions

TOL was separated into supernatant and sediment by centrifugation operation at 12,000 rpm for 10 min. Supernatant (1 ml) was placed into a 5 ml volumetric flask, and 50% HPLC-grade methanol was added to the scale as the supernatant samples.

The separated sediments were washed repeatedly until the cleaning liquid was colorless. After nitrogen blowing drying, 20 ml Dimethyl sulfoxide (DMSO) was added to dissolve it completely as a test solution of sediment.

All standards were accurately weighed and placed in 10 ml volumetric flask and were added 50% HPLC-grade methanol to the scale to produce 25 μg·ml^−1^ GA, 132 μg·ml^−1^ CR, 36 μg·ml^−1^ GC, 20 μg·ml^−1^ C, 56 μg·ml^−1^ ECG, 92 μg·ml^−1^ EGCG, 62 μg·ml^−1^ GCG, 92 μg·ml^−1^ EGC, 116 μg·ml^−1^ EA, 104 μg·ml^−1^ CA, and 42 μg·ml^−1^ CLA standards solutions. Appropriate amount of DPPH was weighed accurately and added absolute ethanol to prepare 0.1 mmol/l DPPH solution. All solutions above were filtered through 0.22 μm membranes (Jinteng, Tianjin, China) before injection.

### Determination of Antioxidant Activity (Jiang et al., 2018)

Appropriate amount of DPPH was weighed out and added to the absolute ethanol. Appropriate amount of DPPH solution was prepared. Then, the supernatant samples were diluted to six different concentrations. Each one with 0.1 ml was added to 3 ml DPPH solution. The mixed solutions kept in a water bath at 40°C for 30 min (in the dark) to guarantee the completeness of the reaction. The absorbance (*A_S_*) at 516 nm was recorded using TU-1810 PC UV-Vis Spectrophotometer (Beijing Persee Corporation, Beijing, China). The absorbance (*A_0_*) of 0.1 ml pure water added to 3 ml DPPH solution was also measured at the same time. The DPPH free radical scavenging rate of the sample was computed as in the following equation:

$$\mathrm{DPPH free radical scavenging rate}(\%)=\frac{A_{0}-A_{S}}{A_{0}}\times 100\%$$

An equation between scavenging ability and concentration was established to calculate *IC_50_*. There is negative correlation between the value and antioxidant activity. The lower the value is, the higher the antioxidant activity is. DPPH assay was used to demonstrate the stability of free radical scavenging activity of solution itself, not used to reflect the TOL used has antioxidant activity in a pharmacological context.

### Turbiscan Lab Expert Conditions

Turbiscan Lab Expert was applied for physical stability studies based on the colloidal properties of TOL. It tended to simultaneously acquire physical parameters like backscattering (BS), transmission (T), turbiscan stability index (TSI), and particle size. TSI was a specific parameter to compare and characterize the physical stability of samples. Any destabilization phenomenon happened would have an effect on BS or transmission signal intensities during the aging process. When TSI went up, the solution system became unstable.

Samples was scanned every 5 h at 880 nm light source in 90 days. Scanning height was 50 mm, waiting time was 20 min, and test temperature was set as 25°C. Optical parameters were set as follows: continuous phase light transmission intensity *T_0_* = 99.99% (water), disperse phase refractive index *n_p_* = 1.36, continuous phase refractive index *n_f_* = 1.33. The remaining parameters were set to default values.

### HPLC Conditions

Samples were analyzed by an Shimadzu LC-20AT high performance liquid chromatography (Shimadzu corporation, Kyoto, Japan) using a Welchrom C_18_ column (4.6 × 250 mm, 5 μm, Shanghai Yuexu Material Technology Co, Ltd., China). Column temperature was 25°C and 10 μl of the sample solution was injected into the system. Mobile phase was composed of (A) 0.1% formic acid in Milli-Q water and (B) methanol using a gradient program of 5% of B at 0–6 min, 5–7% of B at 6–15 min, 7–15% of B at 15–20 min, 15–21% of B at 20–25 min, 21–22% of B at 25–31 min, 22% of B at 31–41 min, 22–28% of B at 41–47 min, 28–32% of B at 47–51 min, 32–38% of B at 51–57 min, 38–45% of B at 57–70 min, and 45–65% of B at 70–80 min. The detection wavelength was 270 nm, with a mobile flow rate of 1 ml min^−1^ (Jiang et al., 2017).

### SEM and Optical Microscope Conditions

A suitable amount of dry sediment was taken and fixed on a sample stage with a double-sided conductive adhesive. After gold-plating treatment, samples were observed using a SEM (SU3500 type scanning electron microscope, Hitachi, Japan). Accelerating voltage of the instrument was 15 kV, working distance was 4.8–5.0 mm, and emission current was 130–135 mA. DM2500 microscope (Leica, Germany) equipped with DP73 type CCD image acquisition and computer image analysis system was applied to complete ordinary microscopic observations.

### ICP-Mass Conditions

Samples were digested using a microwave digestion system (WX-8000, PreeKem Scientific Instruments Co., Ltd., Shanghai, China), equipped with PTFE cans. TOL (0.5 ml) was accurately taken and placed into microwave digestion system, added 5 ml nitric acid, and digested as set parameters. Microwave digestion process was as follows: initially, temperature rose to 120°C within 3 min and maintained at 1800 W for 10 min. Then, temperature increased to 150°C within 5 min and maintained at a power of 1800 W for 10 min. Eventually, temperature went up to 180°C for 30 min at 1800 W.

Metal ions were analyzed by an inductively coupled plasma mass spectrometry (ICP-MS, 7700X, Agilent Technologies, California, USA). Instrument operation parameters were set as follows: radiofrequency power 1.5 kW, carrier gas flow 1.0 l/min, sampling depth 8.0 mm, peristaltic pump speed 0.1 rps, collision cell helium flow 5.0 ml min^−1^, atomization chamber temperature 2°C.

### Data Processing and Analysis

HPLC data files were imported into the similarity evaluation system for chromatographic fingerprint of TCM software (Chinese Pharmacopoeia Commission, version 2012, Beijing, China). After filtering treatment, the data from each sample were normalized and imported into the software SIMCA-P 13.0 (Umetrics, Umea, Sweden), where multivariate analyses, such as S-plot and PLS-DA, were used for calculation. Turbiscan lab expert data and physical parameters of T, BS, TSI, and particle size were automatic calculated and processed using Turbisoft-Lab software (Formulaction corporation version 2.2.0.82, Toulouse, France).

## Results

### DPPH Radical Clearance Measurement Test and Anti-Fatigue Experiment Results

**Figure 2:**
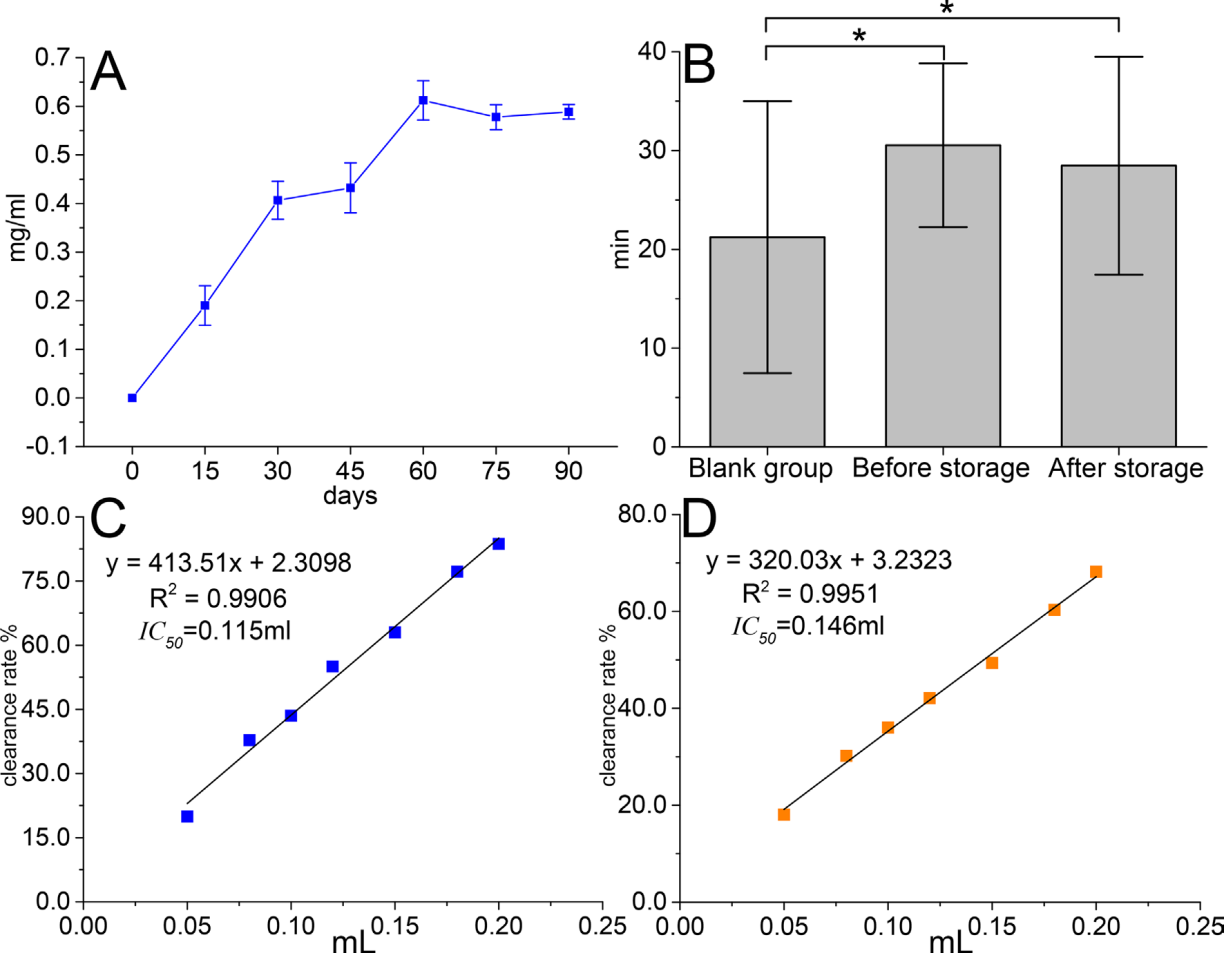
Changes in precipitation amount **(A)**, anti-fatigue result before and after storage **(B)**, antioxidant activity before storage **(C)**, and antioxidant activity after storage **(D)** *p < 0.05.

The results (**Figure 2A**) showed that the amount of sedimentation increased significantly within 3 months and reached 0.6 mg·ml^−1^. Anti-fatigue result (**Figure 2B**) illustrated that TOL intended to prolong swimming time in mice (*p* < 0.05). After 3 months of storage, swimming time tended to decrease, but there is no statistical difference yet. DPPH antioxidant activity test (**Figures 2C, D**) showed that *IC_50_* decreased from 115 μl to 146 μl, which indicated the antioxidant capacity of the supernatant was weakened.

### TSI Index and Colloidal Particle Size Tracking Results

**Figure 3:**
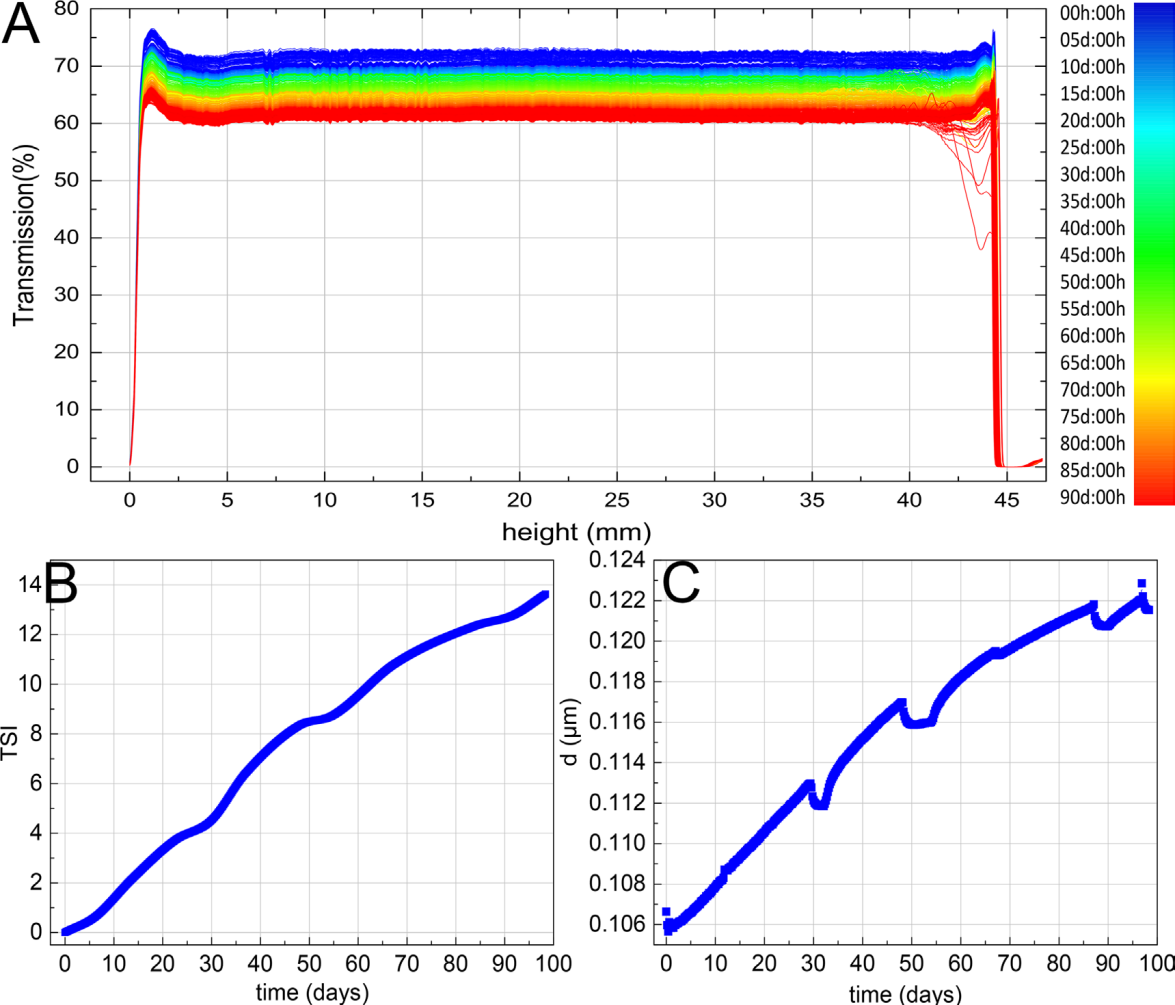
Turbiscan lab expert dynamic monitoring results of TOL transmittance **(A)**, TSI **(B)**, and particle size **(C)**.

The overall light transmittance of solutions reduced from 70% to 60% in 90 days (**Figure 3A**), which suggested the particle size of solution was continuously increasing during storage and the stability was continuously decreased. TSI value rose to 14 (**Figure 3B**), and the average particle size increased from 106 to 122 nm (**Figure 3C**), which indicated that the system was unstable. Based on the data above, the average particle migration rate of solution was 0.24 mm/h. Comparing **Figure 3B** with **Figure 3C**, it was not difficult to infer that particle size and TSI value were highly positively correlated, which demonstrated that the increase of particle size boosts the instability of solution.

### HPLC Fingerprint Analysis Before and After TOL Storage

**Figure 4:**
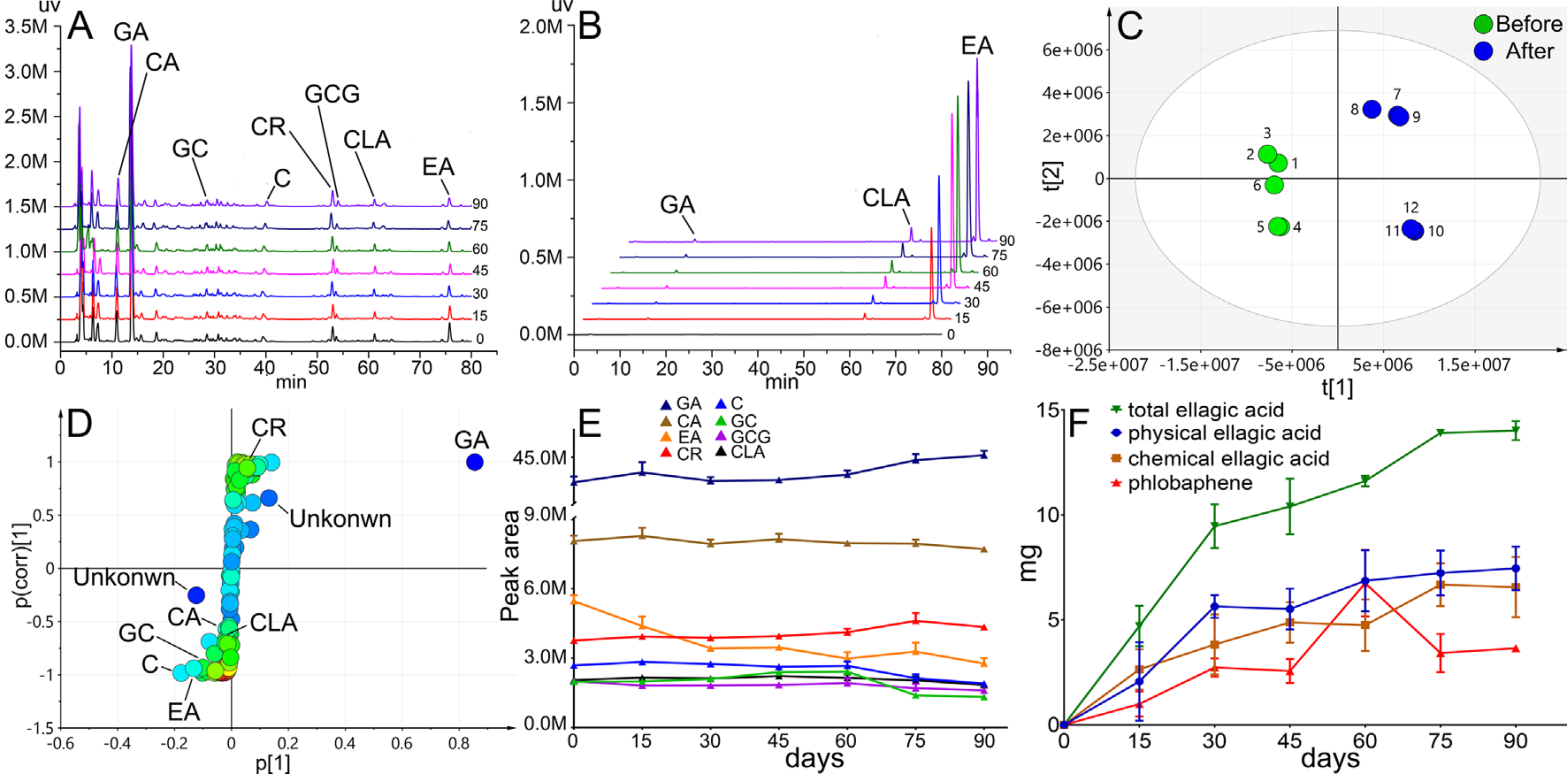
Chemical profile and sedimentation analysis results. HPLC fingerprint of supernatant **(A)** and sediment **(B)**, scatter plots **(C)** and S-plot **(D)** of PLS-DA, trends of chemical markers **(E)**, and trends of ellagic acid and phlobaphene **(F)** during the storage of 90 days.

HPLC fingerprints of TOL supernatant and precipitation before and after storage (90 days) were acquired (**Figures 4A, B**). In the supernatant, 120 peaks were separated well, and chebulic acid, gallic acid, gallocatechin, catechin, corilagin, gallocatechin gallate, chebulagic acid, and ellagic acid were identified. In the sediment, gallic acid, chebulagic acid, and ellagic acid were identified. With the prolongation of storage time, the contents of ellagic acid and chebulagic acid increased sharply. In order to further search for the main chemical markers in the storage process, the peak area data of 120 chromatographic peaks were collected and partial least squares discriminant analysis (PLS-DA) was used. Scatter plots were shown in **Figure 4C**. A clear distinction indicated the migration of chemical constituents before and after storage. Such parameters, the *R^2^*X = 0.826, *R^2^*Y = 0.986, *Q^2^* = 0.966 (*Q^2^* > 0.9), suggested that the model was reliable and has strong internal verification and prediction capability. To explore the potential variables, S-plot was carried out. In S-plot (**Figure 4D**), the farther the variable deviates from the origin, the higher the value of the variable importance plot obtains. Thus, the potential chemical markers that distinguish the samples were discovered in those significantly deviate from the originals. The most obvious chemical marker was identified as ellagic acid (Rt = 13.952) farthest from the origin. Other identified markers included catechin (Rt = 39.447), ellagic acid (Rt = 75.916), gallocatechin (Rt = 28.572) and corilagin (Rt = 53.034), chebulagic acid (Rt = 61.482), and chebulic acid (Rt = 10.906). It was also found that with the sediment increase, the content of ellagic acid, catechin, gallocatechin, gallocatechin gallate, chebulagic acid, and chebulic acid reduced during storage, while that of gallic acid and corilagin increased remarkably (**Figure 4E**). Further tracking the transfer of ellagic acid in supernatant and precipitation, it was a surprise to find that the new-born ellagic acid in precipitation was about 14.02 mg, while the lost in supernatant was only 7.46 mg after 90 days of storage (**Figure 4F**). It indicated that the source of ellagic acid in precipitation was not single, besides colloidal aging; it was also accompanied by chemical degradation. Such hydrolyzed tannins, such as chebulic acid, might be the precursor to ellagic acid (Yoshida et al., 1980; Mao and Wu, 2016). Another interesting finding was the decrease of catechins. It is known that catechins are the precursors for the synthesis of condensated tannins. Phlobaphene is the oxidative polymerization product of condensed tannins. We can speculate that the loss of catechins is involved in the formation of phlobaphene. Due to phlobaphene is a generic term for a class of compounds with many structural types and complexities, it is hard to detect in HPLC (Yassin et al., 2015). Therefore, more evidence of phlobaphene in sediments is needed.

### Microscopic Observation Result

**Figure 5:**
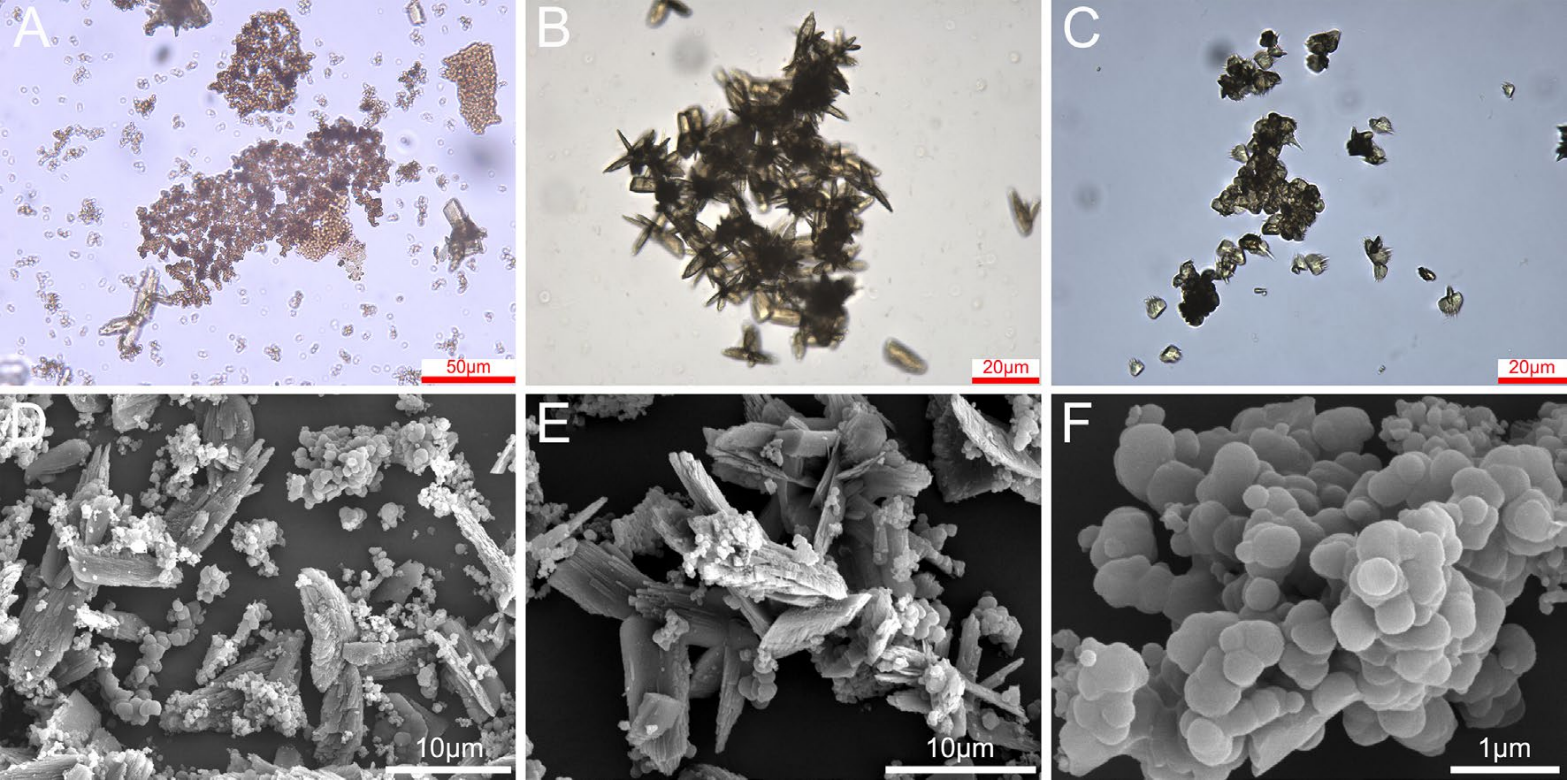
Optical microscope and SEM results of TOL, two sedimentation mixtures **(A** and **D)**, irregular sediment **(B** and **E)**, and crystalline sediment **(C** and **F)**.

It was clear that the sediment (stored for 90 days) mainly included two forms in microscopy (**Figure 5A**). One was a transparent and regular crystal substance (**Figure 5C**), and the other was irregular substance (**Figure 5B**). This result indicates preliminarily that the sediment contained multiple forms and generated by different pathways.

For more surface information, SEM was used to observe the surface structure (**Figure 5D**). The same results as those of the optical microscope were obtained (**Figures 5E, F**). According to previous knowledge, ellagic acid monomer is a crystalline substance (Mathieson and Poppleton, 2010), and phlobaphene is irregular. So, it can be preliminarily speculated that the regular crystal tends to be ellagic acid and the other is likely to be phlobaphene. But this view needs further verification. In addition, it was found that there are many regular layered structures in the crystal. It might be related to the planar molecular structure of ellagic acid. Since the two types of sediments are difficult to separate for measurement (Uchida et al., 2016), micro-area spectroscopy was applied for the further identification.

### Energy Dispersive Spectrometer Analysis Result

**Figure 6:**
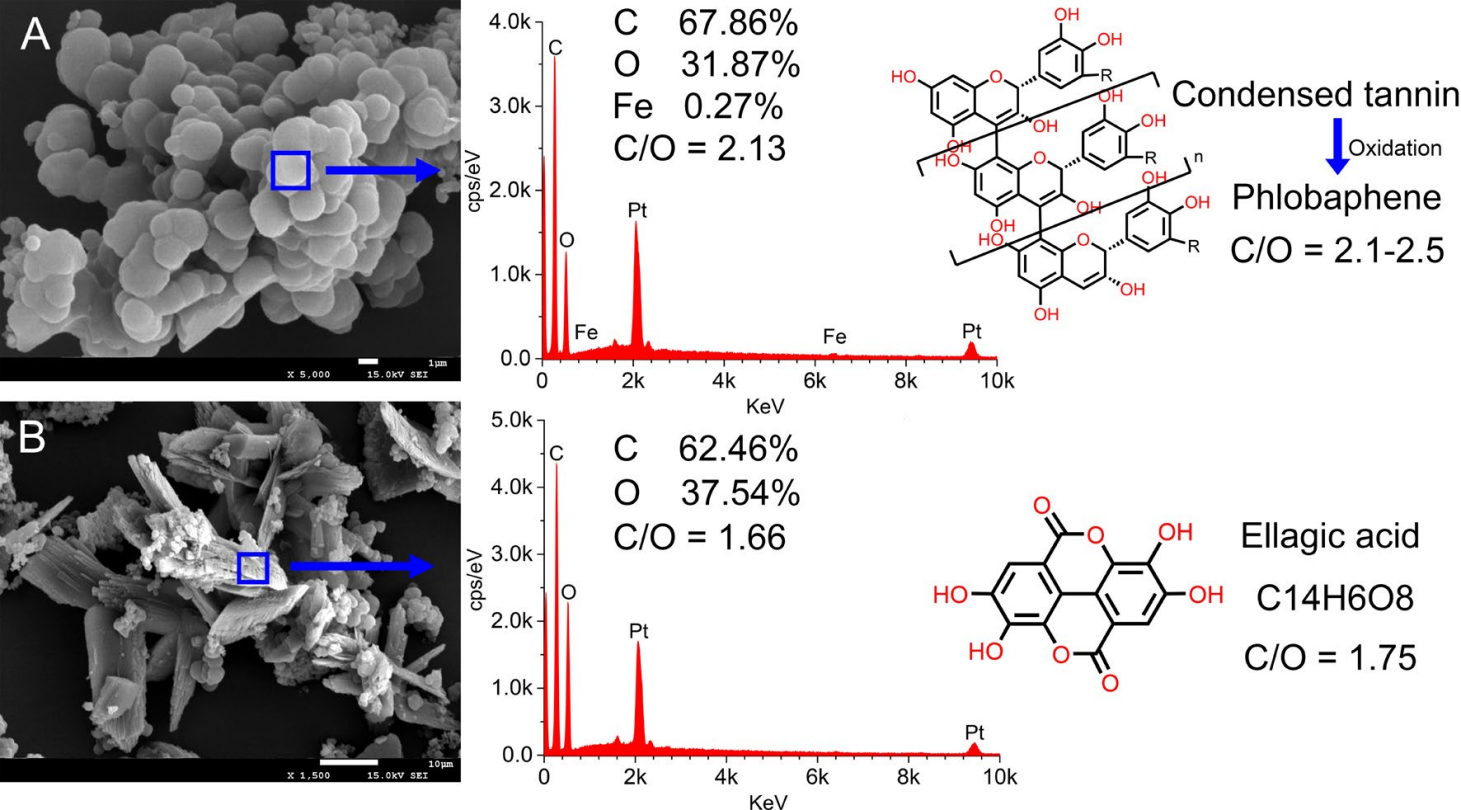
EDS results of multi sedimentation. Irregular sediment **(A)** and crystalline sediment **(B)** elemental scan analysis results.

The results of energy dispersive spectrometer (EDS) were shown in **Figure 6**. The calculation by C/O ratio illustrated that of irregular sediment (**Figure 6A**) was 2.13 and it contained a small amount of iron (0.27%), while that of crystalline sediment (**Figure 6B**) was 1.66 and without iron. Based on theoretical calculation, the C/O ratio of phlobaphene inclined to 2.1–2.5, and that of ellagic acid was supposed to be 1.75. Therefore, it tended to confirm that the irregular sediment was phlobaphene, and the other was ellagic acid. In addition, the existence of Fe ions has attracted our attention. It has been reported that Fe ions can catalyze the formation of orthoquinone, which greatly accelerates the oxidative condensation of catechin to phlobaphene (Uchida et al., 2016). This is another important evidence to confirm tannin precipitation.

### ICP-Mass Results

It was found that the TOL contained 14 elements, such as B, Na, Mg, Al, K, Ca, Ti, Mn, Fe, Ni, Zn, Se, Sr, and Sn. Among them, the contents of Na, Mg, Al, K, and Ca were relatively higher, followed by Fe and other elements. Especially the highest content of K, which might be related to added pharmaceutical excipient of acesulfame potassium. A standard curve was further used to quantitatively determine the content of Mg, Ca, and Fe (**Table 1**). These ions have an influence on the stability of the associated colloids and the polymerization of catechins.

**Table 1:** Quantitative determination of three metal ions in TOL (μg·ml^−^^1^).

Batch		Ca^2+^	Mg^2+^	Fe^3+^
S1		295.56	150.23	6.62
S2		269.26	144.53	4.34
S3		260.66	150.18	4.55

## Discussions

For the sediment of polyphenol solutions, traditional view has always been convinced to be phlobaphene, the oxidation and polymerization products of tannins. However, our study confirmed that phlobaphene and ellagic acid both existed in sediments through an integrated chain of evidence analysis based on the physical phase, chemical profile, and sediment elements. How do these two kinds of sedimentation occur?

It was observed that there were various catechins in TOL, including catechin, gallocatechin, and gallocatechin gallate. Their content gradually decreased in storage, especially after 60 days. In fact, the loss of catechins is involved in the formation of phlobaphene. The main route is “catechins-condensed tannins (dimer and polymer)-phlobaphene.” It means that the two molecules of catechins are slowly polymerized into dimers and polymers in acidic environment. With the increase of polymer molecules, the water solubility gradually decreases until precipitation is formed. During this process, Fe^3+^ plays a key role in the non-enzymatic oxidation of catechins. It was reported that when the concentration of Fe^2+^, Fe^3+^, and Ca^2+^ reached 20, 20, 5, and 200 mg/kg in running water, the catechins can be reduced (Sang et al., 2004; Uchida et al., 2016). In the study, the scanning of Fe^3+^ in irregular sediments and proximity C/O ratio are the direct evidence of the formation of phlobaphene.

Ellagic acid is extremely difficult to dissolve in pure water, and its solubility is only 9.7μg·ml^−1^ (Bala et al., 2006). But the previous study (Jiang et al., 2017) and this experiment both proved that there were excess ellagic acid (more than 0.5 mg·ml^−1^) existed in TOL supernatant, which meant the solubility of ellagic acid in TOL is about 51 times higher than that in pure water. What is the solubilization mechanism behind it? Hundreds of polyphenols can be self-assembled to form an associative colloid with solubilization function. It is a special structure that the hydrophilic end of molecules is outward, and the hydrophobic end is toward the inside. It provides a natural carrier for the solubilization of ellagic acid. Nevertheless, the associated colloid belongs to thermodynamically unstable systems, which is easily influenced by pH, temperature, metal ions, and other factors. When temperature or pH decreases, the intramolecular hydrogen bonds are formed in large quantities, and the association degree increased. High valence metal ions affect the stability mainly by wrecking the electrokinetic potential of colloids. It is thus when the association colloid is destroyed, ellagic acid precipitates without colloid protection.

**Figure 7:**
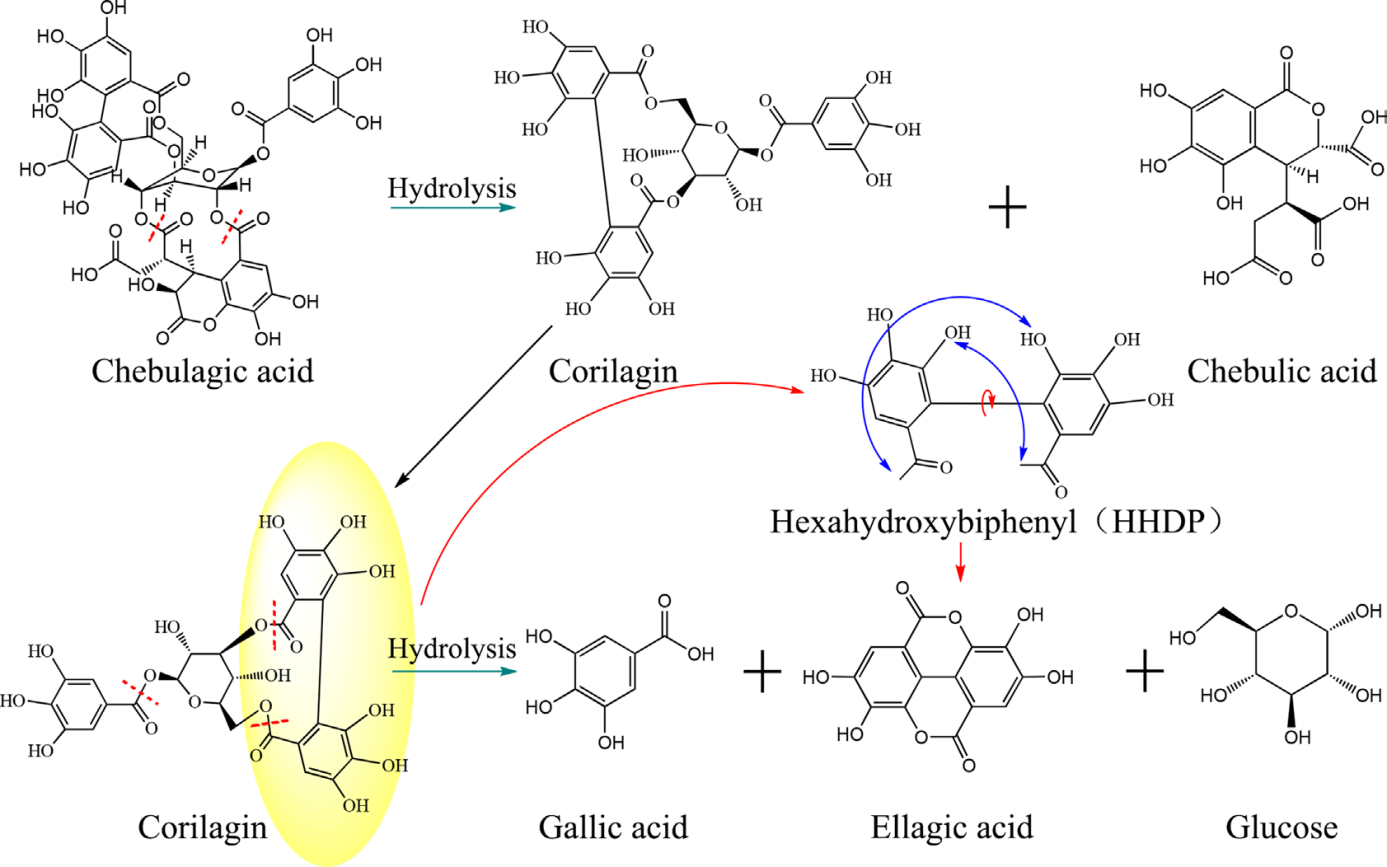
Schematic diagram of the hydrolysis reaction and process of chebulagic acid and corilagin in TOL. The chebulagic acid is first hydrolyzed to form corilagin, and corilagin is further hydrolyzed into ellagic acid and gallic acid.

Does ellagic acid in precipitation originate entirely from physical precipitation? We found an interesting phenomenon that the content of ellagic acid in precipitation was significantly higher than that lost in supernatant. In addition to the physical precipitation caused by colloid destruction, there were other ways to produce ellagic acid. Combining with the change trend of chemical markers, such as chebulagic acid, chebulic acid, and excess ellagic acid, was most likely due to slow hydrolysis of hydrolyzed tannins. Taking chebulagic acid as an example, it was first hydrolyzed into corilagin and chebulic acid under acidic conditions. Then, corilagin was further transformed into gallic acid and hexahydroxybiphenyl (HDDP), and the latter reacted with intramolecular dehydration to form ellagic acid. The possible hydrolysis process is listed in **Figure 7**.

**Figure 8:**
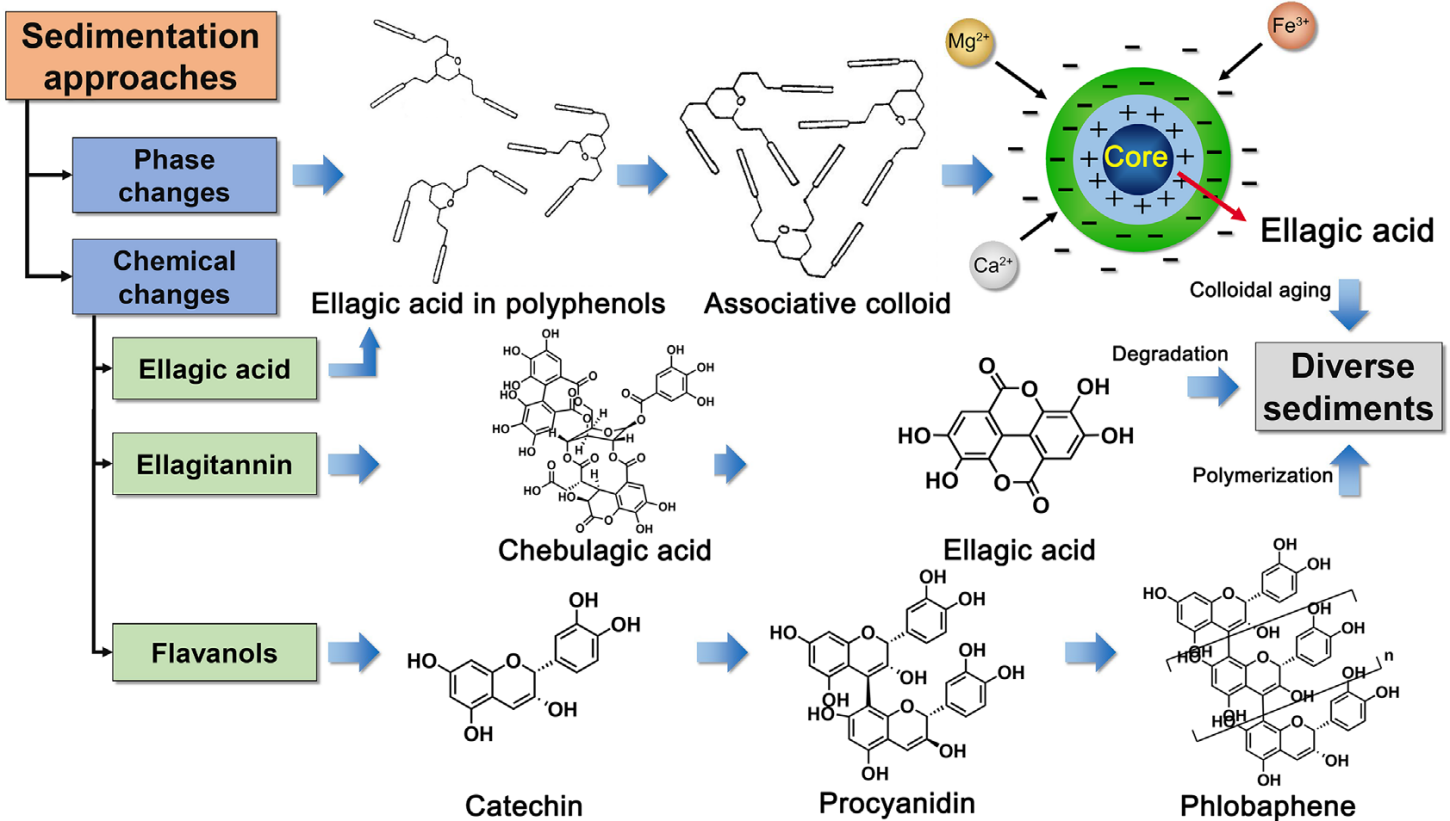
Schematic diagram of main precipitation approaches, including phase change, ellagitannin hydrolysis, and oxidative polymerization of catechins.

The precipitation mechanism of TOL is a valuable research. Two precipitates and three pathways are discovered in the polyphenol solution during storage. Phlobaphene is formed by non-enzymatic polymerization of catechins, while the precipitation of ellagic acid includes physical settlement and chemical degradation. The multiple paths of precipitation are shown in **Figure 8**. Catechins and ellagic acid are the main substances involved in the formation of precipitation. Catechins are reported many functions, including anti-cancer effect (Yang et al., 2009), protection of cardiovascular system (Basu and Lucas, 2007), anti-inflammatory (Shapiro et al., 2009), and anti-fatigue (Yu et al., 2010). Ellagic acid is often regarded as an antioxidant and anti-cancer component (Wang et al., 2012). It is also a potential blood coagulant, which enables to activate factor XII and release kinin in blood. When these ingredients are polymerized or precipitated, the corresponding effects are also weakened. Therefore, it is particularly necessary to find a reasonable solution based on the multiple precipitation paths. The control strategy not only needs to protect colloid stability but also inhibits chemical hydrolysis and polymerization.

This paper reveals three ways for the formation of TOL sedimentation, but it is not known whether these ways are interrelated and which way triggered the sedimentation. Therefore, further research is needed to clarify the micro-mechanism of sedimentation formation so as to restrain sedimentation from its source. In conclusion, this work presents a novel viewpoint on the mechanism of instability of polyphenolic solutions from the perspective of physical and chemical stability. The multiple forms and multiple formation pathway of sedimentation were found based on the evidence chain analysis. It provides a scientific analysis method based on evidence chain. Meanwhile, it also lays an important theoretical foundation for improving the stability of polyphenols preparations, health products, and foods.

## Ethics Statement

This study was carried out in accordance with the recommendations of the Guidelines for the Care and Use of Laboratory Animals of the Ministry of Science and Technology of China. The animal protocol was approved by the Committee on the Ethics of Animal Experiments of Affiliated Hospital of Chengdu University of Traditional Chinese Medicine (Approval ID: 2018BL-002).

## Author Contributions

H-ZH, BF, D-KZ, and LH conceived and designed the study. H-ZH, S-YZ, BF, and J-ZL performed the experiments. H-YM, R-CX, S-HF, H-YL, and Z-FW provide samples, guidance, and suggestions for the experiment. H-ZH, D-KZ, and BF wrote the paper.

## Funding

This study was supported by grants from the General Financial Grant from the China Postdoctoral Science Foundation (2017M623308XB) and Science and technology development fund of Chengdu University of TCM (ZRQN1775, ZRYY1717).

## Conflict of interest Statement

H-YL and S-HF were employed by Sanajon Pharmaceutical Group. The remaining authors declare that the research was conducted in the absence of any commercial or financial relationships that could be construed as a potential conflict of interest.
